# Supplementary material for: Content-rich biological network constructed by mining PubMed abstracts
Source: BMC Bioinformatics. 2004 Oct 8;5:147. doi: 10.1186/1471-2105-5-147 (PMC528731; doi:10.1186/1471-2105-5-147)
Supplement: Additional File 2 — The original results of the above study (non-essential files are deleted to keep the file size under the limit set by BMC bioinformatics). [file 1471-2105-5-147-S2.bz2 › chilibotAdditionalFile2/dip10/270ID2169352E1090/html/NR3C1_JUN.html]

 


 **NR3C1** and **JUN** 
  
Found 213 abstracts in PubMed, retrieved 10.  
 

 What does Google say? 
 PDF only 
| .edu only 

---

**Interactive relationship** (e.g. stimulation, inhibition, etc)

**Inhibitory relationship**- Receptor Interacting Protein 140 Binds c  **Jun**  and Inhibits Estradiol Induced Activator Protein 1  [ **JUN** ]  Activity by Reversing Glucocorticoid Receptor  [ **NR3C1** ]  Interacting Protein 1 Effect.  Ref: 12554755 Mol Endocrinol, 2003
- Regardless of the AP 1  [ **JUN** ]  complex s composition, glucocorticoid receptor  [ **NR3C1** ]  activation inhibits AP 1  [ **JUN** ]  and prevents apoptosis.  Ref: 12766089 Invest Ophthalmol Vis Sci, 2003
**Neutral relationship**- We have previously shown that induction is mediated by the direct interaction between c  **Jun**  and ERalpha, which stabilizes a multiprotein complex containing the coactivator GRIP1 glucocorticoid receptor  [ **NR3C1** ]  interacting protein 1.  Ref: 12554755 Mol Endocrinol, 2003

**Non-interactive relationship** (e.g. studied together, co-existance, homology, etc.)

- The elements likely for nuclear receptors retinoic acid receptor, retinoic acid X receptor, hepatocyte nuclear factor 3, glucocorticoid receptor  [ **NR3C1** ] , nuclear factor kappaB and activator protein 1  [ **JUN** ]  were also found.  Ref: 12384299 Gene, 2002
- These results suggest that AP 1  [ **JUN** ]  proteins and glucocorticoid receptor  [ **NR3C1** ]  regulate transcription of the GnRH R promoter in a heterologous system.  Ref: 12644302 Mol Cell Endocrinol, 2003
- Expression of c fos, rather than c  **jun**  or glucocorticoid receptor  [ **NR3C1** ]  mRNA, correlates with decreased glucocorticoid response of peripheral blood mononuclear cells in asthma.  Ref: 12400872 Int Immunopharmacol, 2002
